# Evolution of ceftazidime-avibactam and cefiderocol resistance in ST131-H30R1-*Escherichia coli* isolates with KPC-3 mutants and application of FTIR biotyping

**DOI:** 10.1128/spectrum.02776-23

**Published:** 2024-02-28

**Authors:** Juan Antonio Castillo-Polo, Marta Hernández-García, Ainhize Maruri-Aransolo, Carmen de la Vega, Patricia Ruiz-Garbajosa, Rafael Cantón

**Affiliations:** 1 Servicio de Microbiología, Hospital Universitario Ramón y Cajal and Instituto Ramón y Cajal de Investigación Sanitaria (IRYCIS), Madrid, Spain; 2 CIBER de Enfermedades Infecciosas (CIBERINFEC), Instituto de Salud Carlos III, Madrid, Spain; Instituto de Higiene, Montevideo, Uruguay

**Keywords:** cefiderocol resistance, ceftazidime-avibactam resistance, KPC mutants, ST131-H30R1-*Escherichia coli*

## Abstract

Ceftazidime-avibactam and cefiderocol represent two of the few alternatives for infections by KPC-producing Enterobacterales. We reported the emergence of resistance to both ceftazidime-avibactam and cefiderocol in a KPC-producing ST131-*Escherichia coli* (KPC-ST131-*Ec*) clinical isolate. Antimicrobial susceptibility testing, Fourier-transform infrared (FTIR) spectroscopy, whole-genome sequencing, and cloning experiments were performed. A KPC-49-*Ec* isolate resistant to ceftazidime-avibactam (MIC_CZA_ > 16/4 mg/L) and susceptible to cefiderocol (MIC_FDC_: 2 mg/L) was recovered in a blood sample from an oncologic patient hospitalized in the medical ICU (June 2019) during ceftazidime-avibactam treatment. After 44 days, a KPC-31-*Ec* resistant to both ceftazidime-avibactam and cefiderocol (MIC_CZA_ > 16/4 mg/L, MIC_FDC_: 8 mg/L) was found in a rectal sample during a second cycle of ceftazidime-avibactam treatment. Both KPC-49 (R163S) and KPC-31 (D179Y) were detected in the epidemic ST131-H30R1-*Ec* high-risk clone and showed a phenotype resembling that of ESBL producers. FTIR spectroscopy managed to differentiate cefiderocol-susceptible and resistant ST131-*Ec* isolates, and these from others belonging to different clones. After cloning and transformation experiments, KPC-49 and KPC-31 were responsible for ceftazidime-avibactam resistance (MIC_CZA_ > 16/4 mg/L) and decreased carbapenem MICs (MIC_MER_ ≤ 0.12 mg/L, MIC_IMI_ ≤ 1 mg/L). KPC-31 was also shown to be associated with increased MICs of cefiderocol (twofold and threefold dilutions over KPC-3 and KPC-49, respectively). However, mutations in proteins participating in outer membrane stability and integrity, such as TolR, could have a more relevant role in cefiderocol resistance. The effects of ceftazidime-avibactam and cefiderocol co-resistance in clinical isolates of Enterobacterales producing KPC mutants make their identification challenging for clinical laboratories.

**IMPORTANCE:**

Throughout four admissions in our hospital of a single patient, different KPC-3 variants (KPC-3, KPC-49, and KPC-31) were found in surveillance and clinical ST131-*Escherichia coli* isolates, after prolonged therapies with meropenem and ceftazidime-avibactam. Different patterns of resistance to cefiderocol and ceftazidime-avibactam emerged, accompanied by restored carbapenem susceptibility. The inability to detect these variants with some phenotypic methods, especially KPC-31 by immunochromatography, and the expression of a phenotype similar to that of ESBL producers, posed challenge to identify these variants in the clinical microbiology laboratory. Molecular methods and whole-genome sequencing are necessary and new techniques able to cluster or differentiate related isolates could also be helpful; this is the case of Fourier-transform infrared spectroscopy, which managed in our study to discriminate isolates by cefiderocol susceptibility within ST131, and those from the non-ST131 ones.

## INTRODUCTION

Cefiderocol is a novel siderophore cephalosporin recently approved by the Food and Drug Administration (FDA) and the European Medicines Agency (EMA) for the treatment of complicated infections caused by MDR Gram-negative organisms (1, 2). Cefiderocol shows activity against a wide range of clinically relevant carbapenemases, including KPC and OXA serine-β-lactamases and VIM, NDM, and IMP metallo-β-lactamases (3, 4). Cefiderocol MIC varies among *Klebsiella pneumoniae* isolates harboring KPC variants that confer resistance to ceftazidime-avibactam with lower outer membrane permeability (5–7). In *Escherichia coli* (Ec), resistance to cefiderocol has also been reported due to a combination of iron transporters deficiency and NDM enzyme production (8, 9).

The ST131-*Ec* high-risk clone (ST131-*Ec*-HRC) is one of the most successful global MDR Gram-negative clones causing human infections (10, 11). The fluoroquinolone resistance-associated H30 sublineage (subclade C1/H30R1 linked to CTX-M-27 and subclade C2/H30Rx linked to CTX-M-15) represent the most prevalent clonal subgroup within the epidemic ST131-*Ec*-HRC (12). Carbapenems are usually chosen to treat infections by ESBL-producing Enterobacterales such as the ST131-*Ec*-HRC; however, it frequently leads to the selection of carbapenemases and thus therapeutic failure (10, 13). Nevertheless, ST131-*Ec-*HRC clinical isolates have shown high percentages of susceptibility to both ceftazidime-avibactam and cefiderocol, so that they could be used to treat infections caused by these bacteria when treatment options are limited (14).

Fourier-transform infrared (FTIR) spectroscopy is a novel typing method with an ability to discriminate similar to PFGE but still lower than whole-genome sequencing (WGS). However, it requires less expertise and is cheaper and faster than WGS analysis (15). The technique compares different spectra obtained by quantification of the infrared light absorption by bacterial cell molecules. Besides this, it is possible to prospectively add samples to the collection and compare them with previous ones, building a repository; however, experience with FTIR in outbreak investigations is still limited (16).

This study presents the development of resistance to both ceftazidime-avibactam and cefiderocol during a treatment with ceftazidime-avibactam in a patient infected and colonized with an ST131-*Ec* isolate producing different KPC-3 mutants. Moreover, the application of FTIR to establish the relationship among *E. coli* isolates was also investigated.

## RESULTS

### Case report

**Fig 1:**
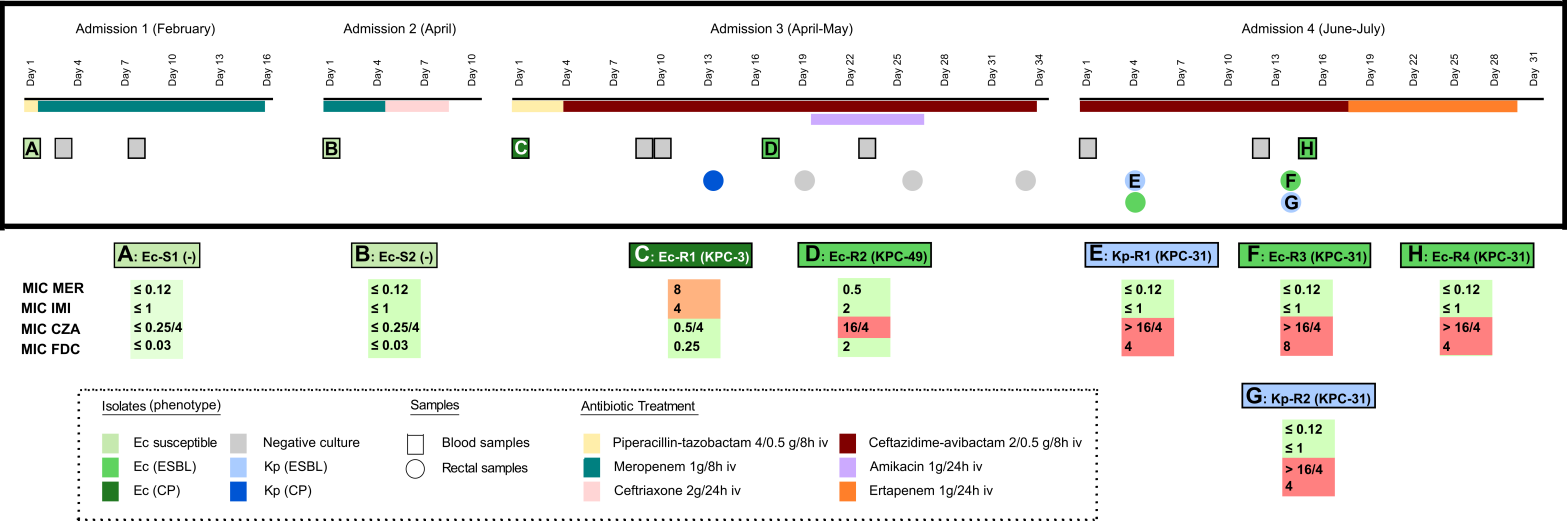
Timeline of isolates and antibiotic treatments* received by the patient during the study period, showing the observed phenotype and the KPC variant. Horizontal bars represent the duration of treatments and all isolates recovered for the analysis are marked with capital letters, indicating the origin of the sample with the shape of the mark (square or circle). Isolates C, D, E, F and H were analysed by WGS. *Only antibiotics with activity against *Escherichia coli* are represented in the figure. mer: meropenem, imi: imipenem, cza: ceftazidime-avibactam, fdc: cefiderocol.

A 48-year-old female diagnosed with biliary cholangiocarcinoma was admitted to the Oncology ward of our hospital four times during 2019, always due to fever or bad general condition, and diagnosed later with acute cholangitis. Five different episodes of bacteremia were documented and *Ec* was isolated in all of the blood samples. During the first two stays, meropenem (1 g/8 h iv) was administrated for 14 days and 4 days, respectively, to treat two episodes of *Ec* bacteremia (*Ec*-S1 and *Ec*-S2, MIC_MER_ ≤ 0.12 mg/L, MIC_IMI_ ≤ 1 mg/L; MIC_CZA_ ≤ 0.25/4 mg/L and MIC_FDC_ ≤ 0.03 mg/L) (Fig. 1; Table 1). In the third admission, a KPC-producing *Ec* (KPC-*Ec*) was detected in a blood culture (*Ec-*R1; MIC_MER_ = 8 mg/L, MIC_IMI_ = 4 mg/L; MIC_CZA_ = 0.5/4 mg/L and MIC_FDC_ = 0.25 mg/L) and ceftazidime-avibactam therapy was implemented (2 g/0.5 g/8 h iv). After 10 days, a second KPC-*Ec* isolate (*Ec-*R2) with an ESBL phenotype but resistant to ceftazidime-avibactam was isolated in another blood sample (MIC_MER_ = 0.5 mg/L, MIC_IMI_ = 2 mg/L; MIC_CZA_ = 16/4 mg/L and MIC_FDC_ = 2 mg/L). Due to that, amikacin was added for 7 days (1 g/24 h iv) (Fig. 1; Table 1). KPC-3 and KPC-49 (R163S mutant of KPC-3) were confirmed by PCR and Sanger sequencing in *Ec*-R1 and *Ec*-R2, respectively. Both *Ec*-R1 (KPC-3) and *Ec*-R2 (KPC-49) were identified as KPC producers by the immunochromatography test and the rapid molecular methods (Eazyplex-Superbug-CRE system and Xpert Carba-R assay) (Table 2). Surveillance cultures were all negative for *E. coli* until this moment.

**TABLE 1:** Antimicrobial susceptibility (AST) results in all *E. coli* and *K. pneumonia*e isolates recovered in the patient during the bacteraemia episodes, determined by EUMDROXF panel^*a*^^,*b*^

Sample	KPC allele	MIC (EUMDROXF, mg/L)																FDC MIC (ComASP, mg/L)	FDC (mm)
		AZT	P/T	FEP	C/T	CZA	FDC	IMI	IMR	MER	MEV	ERV	TOB	AMI	FOS	TGC	COL		
A: *Ec*-S1	(−)	≤1	≤4/4	≤1	≤0.25/4	≤0.25/4	≤0.03	≤1	0.25/4	≤0.12	≤0.06/8	0.25	1	≤2	≤16	≤0.5	≤0.5	0.032	27
B: *Ec*-S2	(−)	≤1	≤4/4	≤1	≤0.25/4	≤0.25/4	≤0.03	≤1	0.25/4	≤0.12	≤0.06/8	0.25	1	≤2	≤16	≤0.5	≤0.5	0.032	26
C: *Ec*-R1	KPC-3	>32	>32/4	>16	>8/4	0.5/4	0.25	4	0.25/4	8	≤0.06/8	0.25	>4	≤2	≤16	≤0.5	≤0.5	0.25	25
D: *Ec*-R2	KPC-49	>32	>32/4	16	>8/4	16/4	2	2	0.12/4	0.5	≤0.06/8	0.25	>4	4	≤16	≤0.5	≤0.5	1	20
E: *Kp*-R1	KPC-31	>32	>32/4	16	>8/4	>16/4	4	≤1	0.25/4	≤0.12	≤0.06/8	0.5	>4	≤2	≤16	≤0.5	≤0.5	4	13
F: *Ec*-R3	KPC-31	16	16/4	>16	>8/4	>16/4	8	≤1	0.25/4	≤0.12	≤0.06/8	0.5	>4	4	≤16	≤0.5	≤0.5	4	15
G: *Kp*-R2	KPC-31	>32	32/4	16	>8/4	>16/4	4	≤1	0.25/4	≤0.12	≤0.06/8	0.5	>4	4	≤16	≤0.5	≤0.5	4	13
H: *Ec*-R4	KPC-31	16	16/4	16	>8/4	>16/4	4	≤1	0.25/4	≤0.12	≤0.06/8	0.25	>4	≤2	≤16	≤0.5	≤0.5	4	15

^*a*^ Cefiderocol susceptibility was also determined by ComASP (broth microdilution) and 30 µg disk diffusion.

^*b*^ AZT: aztreonam, P/T: piperacillin-tazobactam, FEP: cefepime, CZA: ceftazidime-avibactam, FDC: cefiderocol, IMI: imipenem, IMR: imipenem-relebactam, MER: meropenem, MEV: meropenem-vaborbactam, ERV: eravacycline, TOB: tobramycin, AMI: amikacin, FOS: fosfomycin, TGC: tigeclycine, COL: colistin.

**TABLE 2:** Sample data and results of phenotypic methods for KPC detection^*a*^.

Sample	Bacterial identification	Admission	Source of infection	CARBA SMART	Rosco Diagnostica CP	DDST ESBL	DDST CP	ICT	Eazyplex system	Xpert Carba-R	KPC allele	ST131 screening
A: *Ec*-S1	*E. coli*	1	Blood	(−)	(−)	(−)	(−)	(−)	(−)	(−)	(−)	(−)
B: *Ec*-S2	*E. coli*	2	Blood	(−)	(−)	(−)	(−)	(−)	(−)	(−)	(−)	(−)
C: *Ec*-R1	*E. coli*	3	Blood	(+)	(+)	(−)	(+)	(+)	(+)	(+)	KPC-3	(+)
D: *Ec*-R2	*E. coli*	3	Blood	(−)	(−)	(+)	(−)	(+)	(+)	(+)	KPC-49	(+)
E: *Kp*-R1	*K. pneumoniae*	4	Rectal	(−)	(−)	(+)	(−)	(−)	(+)	(+)	KPC-31	NA
F: *Ec*-R3	*E. coli*	4	Rectal	(−)	(−)	(+)	(−)	(−)	(+)	(+)	KPC-31	(+)
G: *Kp*-R2	*K. pneumoniae*	4	Rectal	(−)	(−)	(+)	(−)	(−)	(+)	(+)	KPC-31	NA
H: *Ec*-R4	*E. coli*	4	Blood	(−)	(−)	(+)	(−)	(−)	(+)	(+)	KPC-31	(+)

^*a*^ CP: carbapenemases, ESBL: extended spectrum β-lactamase, ICT: immunochromatography, NA: not applicable.

After 1 month, in the fourth admission and during a second cycle of ceftazidime-avibactam treatment given empirically (2 g/0.5 g/8 h iv), a KPC-producing *K. pneumoniae* (KPC-*Kp*) also with an ESBL phenotype but resistant to ceftazidime-avibactam and cefiderocol was found in a surveillance sample (*Kp*-R1; MIC_MER_ ≤ 0.12 mg/L, MIC_IMI_ ≤ 1 mg/L; MIC_CZA_ > 16/4 mg/L and MIC_FDC_ = 4 mg/L). Another rectal sample collected during the same admission was positive for a KPC-*Kp* (*Kp*-R2) and a KPC-*Ec* (*Ec*-R3), 44 days later than the *Ec*-R2 (KPC-49). Both isolates had an ESBL phenotype (MIC_MER_ ≤ 0.12 mg/L, MIC_IMI_ ≤ 1 mg/L) and were resistant to both ceftazidime-avibactam (MIC_CZA_ > 16/4 mg/L) and cefiderocol (*Ec*-R3, MIC_FDC_ = 8 mg/L; *Kp*-R2, MIC_FDC_ = 4 mg/L). After 1 day, a blood culture was positive for another KPC-*Ec* with a similar phenotype (*Ec*-R4; MIC_MER_ ≤ 0.12 mg/L, MIC_IMI_ ≤ 1 mg/L, MIC_CZA_ > 16 mg/L and MIC_FDC_ = 4 mg/L). As this was the first clinical positive sample in this admission, the therapy was changed to ertapenem for 12 days (1 g/24 h iv) (Fig. 1; Table 1). KPC-31 (D179Y mutant of KPC-3) was confirmed by PCR and Sanger sequencing in all KPC-*Ec* and KPC-*Kp* recovered during this fourth admission, in the second cycle of ceftazidime-avibactam treatment. None of these KPC-31-producing isolates were identified as a KPC carbapenemase producer by the immunochromatography test (Table 2).

All but one of the cefiderocol susceptibility testing results had similar interpretations between disk diffusion and broth microdilution techniques (EUMDROXF and ComASP) (Table 1). The antibiotics and dosages received during the admissions are summarized in Table S1.

### **ST131 screenin**g and Biotyper analysis

PCR screening for the ST131 high-risk clone was positive for all *Ec* isolates recovered from clinical and surveillance samples during the third and fourth admissions, all being KPC producers. The susceptible *Ec* isolates (*Ec*-S1 and *Ec*-S2) were typed as non-ST131 (Table 2).

**Fig 2:**
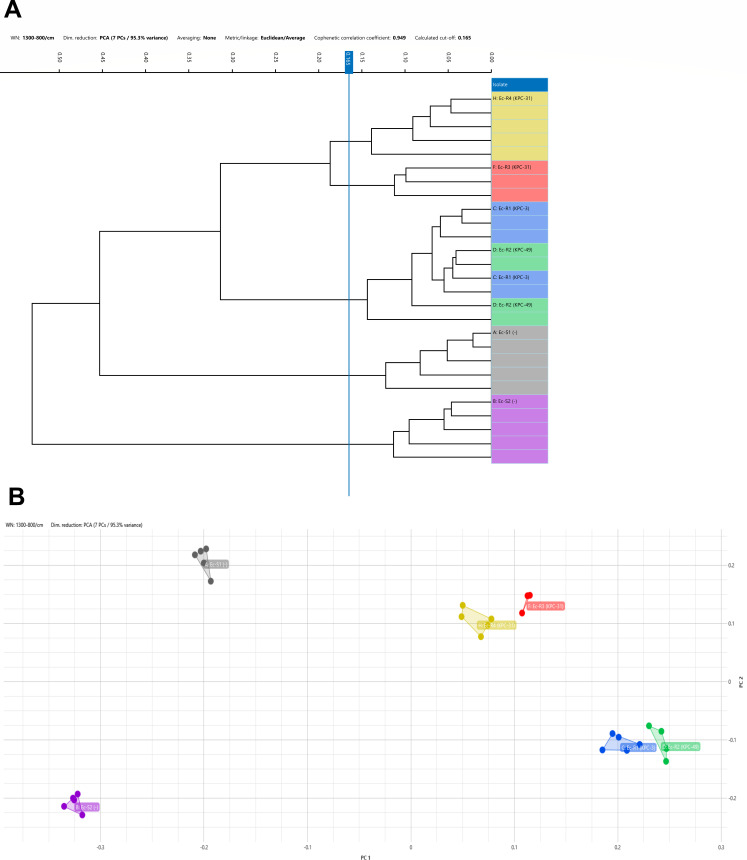
(A) Dendrogram and (B) scatter plot obtained from Fourier-transform infrared spectroscopy (FTIR). Default cut-off: 0.165. Non-ST131 *Ec*-S1 (purple) and *Ec*-S2 (gray) are separated from the ST131 isolates, being differentiated the cefiderocol-susceptible (*Ec*-R1 and *Ec*-R2, KPC-3 and KPC-49, blue and green) and cefiderocol-resistant ones (*Ec*-R3 and *Ec*-R4, KPC-31, red and yellow) within the ST. All isolates were evaluated in quintuplicate, but only the determinations whose quality controls were valid were included (four for *Ec*-R2 and three for *Ec*-R3).

Using the cut-off proposed by the FTIR or Biotyper software (0.165), the cefiderocol-resistant isolates (KPC-31-*Ec-*R3 and KPC-31-*Ec*-R4) were classified very close, whereas susceptible isolates (KPC-3-*Ec-*R1 and KPC-49-*Ec*-R2) were separated from these two isolates in the same cluster, belonging all of them to ST131 clone. Apart from that, non-ST-131 susceptible *E. coli* (*Ec-*S1 and *Ec-*S2) were also differentiated from the previous ones. The distribution is represented in a dendrogram (Fig. 2A) and a scatter plot (Fig. 2B).

### Molecular characterization

Both KPC-3-*Ec*-R1 (WIRF00000000) and KPC-49-*Ec*-R2 (WIRG00000000) isolates were confirmed in our previous study as the ST131-H30R1 subclone (serotype O25:H4, phylogroup B2, clade 1) (17). KPC-31-*Ec*-R3 and KPC-31-*Ec-*R4 isolates were also identified as the ST131-H30R1 high-risk clone. *bla*_KPC-31_ was contained on a *Tn4401a* identical to that found in the *bla*_KPC-3_ and *bla*_KPC-49_-producing isolates. An IncFI-FIA-FIB plasmid that belonged to the F1:A2:B20 sequence type was identified in all isolates. In addition to *bla*_KPC-31_, other resistance mechanisms were detected in both *Ec*-R3 and *Ec-*R4 isolates against β-lactams (*bla*_TEM-1_, *bla*_OXA-18_), aminoglycosides [*aac (3)-IIa*, *aadA5*,*aac (3)-IId* and *aph (6)-Id*], macrolides (*mphA*), sulfonamides (*sul1* and *sul2*), tetracycline (*tetA*), and trimethoprim (*dfrA17*). Coinciding with KPC-3-*Ec*-R1 and KPC-49-*Ec*-R2, genes encoding *bla*_CTX-M_ or other carbapenemases were not identified in KPC-31-*Ec*-R3 or KPC-31-*Ec-*R4. Additionally, all strains carried identical known mutations in the quinolone resistance-determining region (QRDR) [*gyrA* (S83L, D87N), *parC* (S80I, E84V), and *parE* (I529L) genes].

Variant calling was performed in KPC-49-*Ec*-R2, KPC-31-*Ec*-R3, and KPC-31-*Ec-*R4 isolates using the KPC-3-*Ec*-R1 as the reference genome. Phylogenetic comparison showed a *core genome* of 5,210,174–5,342,762 pb with a range of SNPs or InDels between 1 and 35 (Fig. S1). Differences were not found in the Average Nucleotide Identity (ANI) value among all isolates (ANI-value = 99.98%). Non-synonymous single-nucleotide polymorphisms (SNPs) were found affecting two genes in KPC-49-*Ec*-R2 (*rpnA*-H82Q, *fdrA*-L433F) and four genes in KPC-31-*Ec*-R3 and KPC-31-*Ec*-R4 (*proQ*-H23fs, *yecH*-A23D, *rfaH*-R73C, and *tolR*-A112V). A mutation in an intergenic region (tRNA-Met, 10310C > G) was also detected in KPC-49-*Ec*-R2, KPC-31-*Ec*-R3, and KPC-31-*Ec*-R4 isolates (Table S2). Mutations in other genes suspected to be possibly involved in the cefiderocol resistance (PBP3-*ftsI*, *cirA*, *fiu*, *fhu*, *fepA*, and *tonB*) were not detected neither in KPC-49-*Ec*-R2 nor in KPC-31-*Ec*-R3 and KPC-31-*Ec*-R4 isolates.

### Cloning results

pKPC-3, pKPC-49, and pKPC-31 recombinant plasmids were successfully obtained by cloning the corresponding *bla*_KPC_ genes. Recombinant plasmids were subsequently transformed by heat shock into isogenic TOP10-*Ec* cells. An increased ceftazidime-avibactam MIC (MIC_CZA_ > 16 mg/L) and a reduction of the MIC values for carbapenems (MIC_MER_ ≤ 0.12 mg/L, MIC_IMI_ ≤ 1 mg/L) was demonstrated in both KPC-49- and KPC-31-TOP10-*Ec* over the corresponding KPC-3-TOP10-*Ec* transformant (MIC_CZA_ = 1 mg/L; MIC_MER_ = 16 mg/L, MIC_IMI_ > 8 mg/L). Regarding the MIC values of cefiderocol, an increase of twofold and threefold dilutions was observed in the KPC-31-TOP10-*E. coli* transformant (MIC_FDC_ = 1 mg/L) over the corresponding KPC-3-TOP10-*Ec* (MIC_FDC_ = 0.25 mg/L) and KPC-49-TOP10-*Ec* (MIC_FDC_ = 0.125 mg/L), respectively (Table S3).

## DISCUSSION

To the best of our knowledge, this is the first description of a KPC-31-producing ST131-H30R1-*Ec* clinical isolate resistant to both ceftazidime-avibactam and cefiderocol. Ceftazidime-avibactam-resistant KPC enzymes usually derive from point mutations in *bla*_KPC-2_ and *bla*_KPC-3_ genes, frequently after the antibiotic exposure (18). Recent publications have demonstrated that the ceftazidime-avibactam and cefiderocol resistance phenotypes and the corresponding resistance mechanisms are commonly correlated, mainly in *K. pneumoniae* (8, 19). However, variable percentages of resistance to both ceftazidime-avibactam (0.5–18%) and cefiderocol (0.9–8%) have been reported in the ST131-*Ec* population (14, 20).

In our study, we reported the emergence of resistance to ceftazidime-avibactam in the ST131-H30R1-*Ec*, first producing the KPC*-*49 (R163S) and then the KPC-31 (D179Y), with a difference in time of 44 days and each of them during a different treatment cycle with ceftazidime-avibactam. Although further studies would be needed to demonstrate this fact, our results suggest that the production of KPC-49 and KPC-31 enzymes in the ST131-*Ec* high-risk clone has possibly occurred through two different routes, since we observed that the genetic distance between KPC-3-*Ec* and KPC-31-*Ec* isolates was smaller than the one between KPC-49-*Ec* and both KPC-31-*Ec*.

On the other hand, co-resistance to cefiderocol was only detected in KPC-31-ST131-*Ec* isolates, even though the MIC value for cefiderocol in the KPC-49-ST131-*Ec* (MIC_FDC_ = 2 mg/L) was threefold higher than that in the KPC-3-ST131-*Ec* isolate (MIC_FDC_ = 0.25 mg/L) and at least sixfold higher than that in the susceptible *Ec* isolates (MIC_FDC_ ≤ 0.03 mg/L), recovered before the initiation of the ceftazidime-avibactam therapy. Hobson *et al*. showed that KPC-31-producing isolates show cross-resistance to both ceftazidime-avibactam (MIC_CZA_ > 32 mg/L) and cefiderocol (MIC_FDC_ = 4 mg/L) (19). Our results demonstrated that both KPC-31 and KPC-49 were responsible for the ceftazidime-avibactam resistance (MIC_CZA_ > 16 mg/L) in our ST131-H30R1-*Ec*. Apart from that, the MIC value for cefiderocol was threefold dilutions higher in the KPC-31-*Ec* transformant (MIC_FDC_ = 1 mg/L) than in the corresponding KPC-3 (MIC_FD_ = 0.25 mg/L) and twofold dilutions higher than in the KPC-49-transformant (MIC_FDC_ = 0.125 mg/L), but still not considered resistant any of them.

In addition to the changes in β-lactamases, a mutation leading an amino acid change in *tolR* (A112V) was identified in both KPC-31-ST131-*Ec*. TolR (homolog of ExbD in the TonB-ExbB-ExbD System), is part of the Tol-Pal system and is required for the maintenance of the outer membrane stability and integrity (21). TonB-ExbB-ExbD system is involved in the active transport of iron siderophores and facilitates the entry of cefiderocol into the bacterial periplasmic space (22, 23). Modifications in TonB-dependent receptors have been demonstrated to be related to cefiderocol resistance in *Pseudomonas aeruginosa* and several Enterobacterales species (24, 25). According to our results, mutations in TolA-Q-R proteins may also inhibit the siderophore-drug uptake and contribute to cefiderocol resistance. Mutations associated with deficiency in iron transporters (*cirA* and *fiu*) and the PBP-3 have also been showed to be implicated in increased cefiderocol MIC values in *Ec* isolates, but identical genes were found in our KPC-3, KPC-49, and KPC-31-*Ec* isolates (5, 6, 9).

Co-colonization with a KPC-31-*K. pneumoniae* isolates resistant to ceftazidime-avibactam and cefiderocol was also detected during the second cycle of treatment with ceftazidime-avibactam. This KPC-31-*Kp* isolate was typed in a previous study as the ST307 high-risk clone, widely disseminated in our hospital since 2018 and linked to the production of other KPC variants conferring resistance to ceftazidime-avibactam (7, 26). Note that co-resistance to ceftazidime-avibactam and cefiderocol has previously been reported in our hospital in KPC-62-producing ST307-*K. pneumoniae* isolates with permeability defects during an outbreak in the medical ICU in 2020 (7). The KPC-31-ST307-*Kp* isolate carried a modification of the PBP2 protein (A523T), possibly also involved in increasing the cefiderocol MIC value, which was not found in any of the *E. coli* isolates (7).

An IncF [F1:A2:B20] plasmid closely related to that found in the ST307-*K. pneumoniae* isolates carrying novel KPC variants previously detected in our hospital were identified in our KPC-3, KPC-49, and KPC-31-*Ec* isolates (7, 26). The presence in circulating *K. pneumoniae* hospital clones of KPC variants conferring resistance to ceftazidime-avibactam and possibly with an impact on the cefiderocol susceptibility, could facilitate the persistence of these resistance mechanisms and their dissemination to other bacterial species such as *Ec*.

Note also that the infection and colonization by ceftazidime-avibactam and cefiderocol-resistant KPC-31-*Ec* and KPC-31-*K. pneumoniae* isolates occurred in this patient before the approval and the use of cefiderocol in clinical practice in our hospital. Besides this, despite of resembling an ESBL phenotype, the immunochromatography test was able to detect the KPC-49 enzymes, but not KPC-31. The detection of cefiderocol resistance in microbiology laboratories represents a considerable challenge due to the technical issues of the phenotypic methods used for the antimicrobial susceptibility testing (27). These limitations, in addition to the successful adaptability of these new KPC variants in high-risk clones with a high capacity for dissemination in the hospital environment, such as the ST131-*Ec*, makes the impact of these findings even more important.

Interestingly, FTIR biotyping managed to differentiate not only the two *Ec* belonging to clones other than ST131 from this one, but also those resistant and susceptible to cefiderocol among ST131 isolates. Changes in the bacterial membrane due to mutations affecting the Tol-Pal system that are believed to contribute to cefiderocol resistance could be the explanation for the ability of this technique to discriminate among them. It should be noted that *Ec*-R3 was obtained from a surveillance rectal swab, whereas the rest of the *Ec* isolates were from blood samples, which might have had an impact on FTIR due to other biochemical features different from KPC changes. FTIR was originally designed to be used as an early warning tool for outbreaks by generating a fingerprint of the bacterial composition and clustering isolates that belong to the same clone; however, more evaluations are needed to fully understand the application in these situations involving similar carbapenemases with different susceptibility results.

In conclusion, our results indicate that mutated KPC β-lactamases are not the only factor responsible for the increased cefiderocol MICs and that mutations affecting genes involved in the energy transduction for the outer membrane integrity, such as the Tol-Pal system, could also contribute to cefiderocol resistance in *Ec*. New biotyping technologies, such as FTIR, might be promising in discriminating mechanisms of resistance involving biochemical changes in the membrane. The production of mutated KPCs affecting cefiderocol susceptibility during treatment with ceftazidime-avibactam in successful high-risk clones such as the ST131-*Ec*, is a public health problem that needs to be monitored and poses a challenge for clinical microbiology laboratories.

## MATERIALS AND METHODS

### Bacterial isolates and patient data

We conducted a retrospective screening of cefiderocol-resistant isolates among the ceftazidime-avibactam-resistant KPC-producing Enterobacterales isolates recovered in our hospital between 2018 and 2020. A KPC-*Ec* resistant to both ceftazidime-avibactam and cefiderocol was found in a surveillance rectal sample from an oncologic patient admitted to our hospital in June 2019. One KPC-*Kp* isolate with the same phenotype was also recovered in a rectal sample in the same period of time in this patient. Clinical and epidemiological data were retrospectively reviewed. All *Ec* and *Kp* isolates that could be recovered in surveillance and clinical samples from this patient were included in the study.

The patient had previously been admitted to our hospital (April 2019) with a bacteremia by a KPC-3-producing *Ec* susceptible to ceftazidime-avibactam (MIC_CZA_ = 0.5/4 mg/L), and a KPC-49-producing *Ec* isolate with a ceftazidime-avibactam resistant phenotype (MIC_CZA_ = 16/4 mg/L), following 10 days of standard-dose ceftazidime-avibactam treatment. The susceptibility testing and molecular characterization of these two KPC-3- and KPC-49-*Ec* isolates were reported in a previous study (17).

### Bacterial identification and susceptibility testing

Rectal samples were directly seeded on ChromID ESBL and ChromID CARBA SMART biplate selective chromogenic agar plates (bioMérieux, Marcy-l’Etoile, France) and incubated at 37°C for 24 or 48 h. Blood cultures were incubated in BACTEC^TM^ FX (BD, USA) and subcultured once flagged positive. Identification of bacteria was performed by mass spectrometry, using MALDI-TOF-MS (Bruker-Daltonics, Bremen, Germany). Antimicrobial susceptibility was assessed by standard broth microdilution using Sensititre EUMDROXF (ThermoFisher, USA). ComASP panel (broth microdilution; Roseto degli Abruzzi, Liofilchem, Italy) and disk diffusion (30 µg disk; Liofilchem) were also used for cefiderocol. MICs and zone diameters were interpreted using EUCAST-2023 criteria (http://www.eucast.org/clinical_breakpoints/).

### KPC carbapenemase detection

Phenotypic detection of KPC carbapenemase was performed using the KPC/MBL/OXA-48 Confirm Kit (Rosco Diagnostica, Taastrup, Denmark) and the immunochromatography test O.K.N.V.I. RESIST-5 (CORIS BioConcept, Gembloux, Belgium). Susceptibility testing by double-disk synergy test (DDST) and ESBL/AmpC Screen Kit tests (Rosco Diagnostica) were also performed. KPC carbapenemase genes were detected by the Eazyplex-Superbug-CRE system (Amplex-Biosystems, Germany) and the Xpert Carba-R assay (Cepheid, Sunnyvale, USA) and then confirmed by PCR and Sanger sequencing.

### Molecular typing

ST131 screening and subtyping to detect the *H30*R1 (clade 1) and *H30*Rx (clade 2) subclones were performed by SNP-based PCR as previously described (28). Bruker’s IR-Biotyper (Bruker-Daltonics, Germany) was also used as biotyping method (https://www.bruker.com/en/products-and-solutions/microbiology-and-diagnostics/microbial-strain-typing.html). *Ec* samples were prepared according to the manufacturer’s instructions, performing five replicates per sample with default settings. The cutoff value proposed by the software was used to determine the cluster aggrupation (16, 29).

### WGS and bioinformatics analysis

Whole-genome sequencing with 2 × 150 pb paired-end reads (Illumina NovaSeq 6000 platform, OGC, Oxford, UK) was performed in all KPC-*E. coli* resistant to ceftazidime-avibactam and cefiderocol detected in this patient (KPC-31-*Ec*-R3 and KPC-31-*Ec-*R4). Both KPC-3-*Ec*-R1 (WIRF00000000) and KPC-49-*Ec*-R2 (WIRG00000000) complete genomes were previously obtained (17) and were also included in the subsequent genetic analysis. Sequencing processing, molecular typing, screening for acquired resistome, and plasmid characterization were carried out as previously described (7, 17). SNPs and small insertions and deletions (InDels) between all isolates were extracted using Snippy 4.6.0, as previously described (17). A phylogenetic tree was constructed using IQtree2 (v.2.0.7) and visualized using iTOL (https://itol.embl.de/). The ANI value was calculated (https://www.ezbiocloud.net/tools/ani) to measure nucleotide-level genomic similarity between two genomes. Specific mutations in genes involved in the resistance to cefiderocol were also analyzed. The complete genome of KPC-31-*Ec*-R3 and KPC-31-*Ec-*R4 isolates were deposited at DDBJ/ENA/GenBank under the project number PRJNA747261.

### *bla*_KPC_ cloning

*bla*_KPC-3_, *bla*_KPC-49_, and *bla*_KPC-31_ genes were amplified by PCR using primers previously described by Shields et al. (18). Amplicons were cloned into the pCR-Blunt^TM^ II-TOPO^TM^ vector following the manufacturer’s instructions (Zero Blunt TOPO PCR cloning kit; Invitrogen, Cergy-Pontoise, France). The recombinant plasmids were transformed by heat shock into competent *Ec* cells (One Shot TOP10 Chemically Competent *E. coli* cells, ThermoFisher) and then selected on Luria broth agar medium supplemented with ampicillin (30 mg/L), kanamycin (50 mg/L), and IPTG-Xgal (80 mg/L), as previously described (17). Successful transfer of *bla*_KPC_ genes was confirmed by PCR and Sanger sequencing. MIC values in KPC-3, KPC-49, and KPC-31 transformants were also measured by standard broth microdilution.

## Data Availability

The complete genomes of sequenced isolates in this project were deposited at DDBJ/ENA/GenBank under the accession numbers: WIRF00000000 (KPC-3-*Ec*-R1), WIRG00000000 (KPC-49-*Ec*-R2), JAHWYX000000000 (KPC-31-*Ec*-R3), and JAZEUX000000000 (KPC-31-*Ec*-R4).

## SUPPLEMENTAL MATERIAL

Supplemental material spectrum.02776-23-s0001.docx Tables S1, S2, and S3; Figure S1.

ASM does not own the copyrights to Supplemental Material that may be linked to, or accessed through, an article. The authors have granted ASM a non-exclusive, world-wide license to publish the Supplemental Material files. Please contact the corresponding author directly for reuse.
