# Supplementary material for: Evolution of ceftazidime-avibactam and cefiderocol resistance in ST131-H30R1-Escherichia coli isolates with KPC-3 mutants and application of FTIR biotyping
Source: Microbiol Spectr. 2024 Feb 28;12(4):e02776-23. doi: 10.1128/spectrum.02776-23 (PMC10986490; doi:10.1128/spectrum.02776-23)
Supplement: Supplemental material — Tables S1, S2, and S3; Figure S1. [file spectrum.02776-23-s0001.docx]

| **Table S1**. Antimicrobials record, including the treatment given to the patient during the admissions. Carbapenems and ceftazidime-avibactam and their durations are in bold. | |
| --- | --- |
| **Admission** | **Antibiotic treatment** |
| 1 | Piperacillin-tazobactam 4/0.5g/8h + vancomycin 1,250 mg/24h IV (1 day) |
|  | **Meropenem 1g/8h** + vancomycin 1,250 mg/24h IV **(14 days)** |
| 2 | **Meropenem 1g/8h** + teicoplanin 400 mg/24h IV **(4 days)** |
|  | Ceftriaxone 2g/24h IV (4 days) |
| 3 | Piperacillin-tazobactam 4/0.5g/8h IV (3 days) |
|  | **Ceftazidime-avibactam 2/0.5g/8h (30 days)** + linezolid 600 mg/12h (24 days) + metronidazole 500 mg/8h (17 days) + amikacin 1g/24h IV (7 days) |
| 4 | **Ceftazidime-avibactam 2/0.5 g/8h (17 days)** + teicoplanin 400 mg/24h IV (7 days) |
|  | **Ertapenem 1g/24h** IV (**12 days)** |

| **Table S2.** SNPs (missense mutations or INDELs) detected in the *core genome* of KPC-49- and KPC-31-producing *E. coli* isolates over the corresponding KPC-3-producing *E. coli.* | | |
| --- | --- | --- |
| **Isolate** | **Mutations** | **Product** |
| Ec-R2 (KPC-49) | *rpnA* (246C>A,H82Q) | Rpn family transposase |
|  | *fdrA* (1299A>T,L433F) | Acyl-CoA synthetase FdrA |
|  | 10310C>G | tRNA-Met |
| Ec-R3 (KPC-31) | *proQ* (63delT, H23fs) | RNA chaperone ProQ |
|  | *yecH* (68C>A, A23D) | Metal-binding protein |
|  | *rfaH* (217C>T, R73C) | Transcription antitermination protein RfaH |
|  | *tolR* (335C>T, A112V) | Colicin uptake protein TolR |
|  | 10310C>G | tRNA-Met |
| Ec-R4 (KPC-31) | *proQ* (63delT, H23fs) | RNA chaperone ProQ |
|  | *yecH* (68C>A, A23D) | Metal-binding protein |
|  | *rfaH* (217C>T, R73C) | Transcription antitermination protein RfaH |
|  | *tolR* (335C>T, A112V) | Colicin uptake protein TolR |
|  | 10310C>G | tRNA-Met |

| **Table S3.** Antimicrobial susceptibility results in TOP10-*E. coli* transformants carrying KPC-3, KPC-49 and KPC-31-recombinant plasmids. | | | | | | | | | | | | | | | | | | |
| --- | --- | --- | --- | --- | --- | --- | --- | --- | --- | --- | --- | --- | --- | --- | --- | --- | --- | --- |
| **Transformant** | **MIC (EUMDROXF®, mg/L)** | | | | | | | | | | | | | | | | **FDC MIC (ComASP®, mg/L)** | **FDC (mm)** |
|  | **AZT** | **P/T** | **FEP** | **C/T** | **CZA** | **FDC** | **IMI** | **IMR** | **MER** | **MEV** | **ERV** | **TOB** | **AMI** | **FOS** | **TGC** | **COL** |  |  |
| TOP10-*E. coli*-KPC-3 | >32 | >32/4 | >16 | >8/4 | 1/4 | 0.25 | >8 | 0.25/4 | 16 | ≤0.06/8 | 0.06 | ≤0.5 | ≤2 | ≤16 | ≤0.5 | ≤0.5 | 0.25 | 26 |
| TOP10-*E. coli*-KPC-49 | 4 | 16/4 | 2 | >8/4 | >16/4 | 0.125 | ≤1 | 0.25/4 | ≤0.12 | ≤0.06/8 | 0.06 | ≤0.5 | ≤2 | ≤16 | ≤0.5 | ≤0.5 | 0.25 | 27 |
| TOP10-*E. coli*-KPC-31 | 1 | ≤4/4 | ≤1 | >8/4 | >16/4 | 1 | ≤1 | 0.25/4 | ≤0.12 | ≤0.06/8 | 0.06 | ≤0.5 | ≤2 | ≤16 | ≤0.5 | ≤0.5 | 1 | 19 |
| AZT: aztreonam, P/T: piperacillin-tazobactam, FEP: cefepime, CZA: ceftazidime-avibactam, FDC: cefiderocol, IMI: imipenem, IMR: imipenem-relebactam, MER: meropenem, MEV: meropenem-vaborbactam, ERV: eravacycline, TOB: tobramycin, AMI: amikacin, FOS: fosfomycin, TGC: tigeclycine, COL: colistin. | | | | | | | | | | | | | | | | | | |


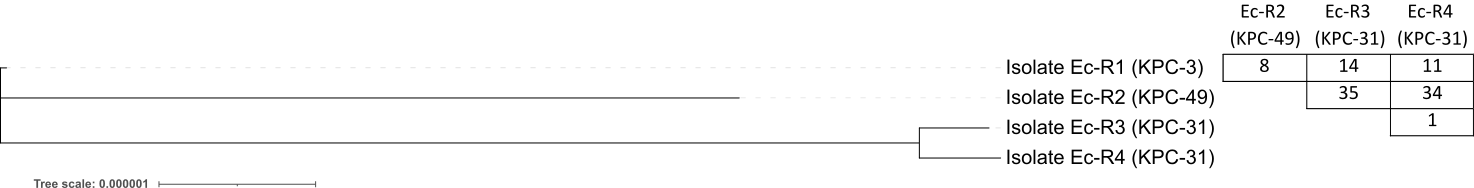


**Figure S1.** Phylogenetic comparison of ST131-*E. coli* isolates producing KPC carbapenemases (KPC-3, KPC-49 and KPC-31) recovered in the patient. Total of variants detected (SNPs and InDels) are also indicated.
